# Clinical profile of COVID-19-associated mucormycosis patients and the clinical suspects: a descriptive audit

**DOI:** 10.1186/s43163-023-00430-2

**Published:** 2023-04-25

**Authors:** Sophia Amalanathan, C Satish Kumar, R Abinaya, K Prathiba, Colbert Kumaran Ramesh, B Kavitha, A Malini, Reddy Amudhasubba

**Affiliations:** 1 Department of ENT, Indira Gandhi Medical College & Research Institute, Pondicherry, India; 2 Department of Medicine, Indira Gandhi Medical College & Research Institute, Pondicherry, India; 3 Department of Microbiology, Indira Gandhi Medical College & Research Institute, Pondicherry, India; 4 Department of Ophthalomology, Indira Gandhi Medical College & Research Institute, Pondicherry, India

**Keywords:** Rhino-orbital-cerebral mucormycosis, Uncontrolled diabetes, Amphotericin B

## Abstract

**Background:**

India witnessed a massive surge of rhino orbital cerebral mucormycosis (ROCM) cases during the second wave of COVID-19, recording the highest number of cases in the world, indeed, an epidemic within the pandemic.

**Objectives:**

To describe the clinical profile of patients with COVID-19-associated mucormycosis (CAM) and the clinical suspects for mucormycosis.

**Methods:**

This single-center descriptive, observational study/audit was done at Indira Gandhi Medical College, Pondicherry, South India. This study is about the clinical profile of 7 CAM patients and 14 COVID-19 patients who were suspects of CAM, based on their risk factors and clinical symptoms, and were referred to the ENT department.

**Statistical analysis:**

All the descriptive variables were summarized as mean, frequency, and percentages for qualitative data.

**Results:**

All 7 CAM patients were COVID-19 positive and were not vaccinated against COVID-19, All 7 were known diabetic, all 7 had steroid therapy for their COVID status, and 5 out of 7 (71%) had uncontrolled diabetes mellitus at the time of diagnosis. Facial pain, nasal discharge, and eye swelling were the presenting symptoms of CAM. Maxillary and ethmoid sinuses were the most commonly involved para nasal sinuses. Four out of seven (57.1%) CAM patients survived after 16 months of follow-up, after surgical and medical treatment for CAM. Of the 14 clinical suspects who were negative for CAM, 2 were negative for COVID-19, their risk factors were brought under control, 3 expired due to COVID complications, and 9 patients are alive till date.

**Conclusion:**

Uncontrolled diabetes is a risk factor for ROCM/CAM, another possible risk factor is steroid therapy, and we hypothesize that COVID infection could also be a possible risk factor that needs to be studied more extensively in a larger sample. Early clinical suspicion, withdrawal of steroids, rapid control of diabetes mellitus, appropriate investigations, and early surgical intervention combined with medical treatment offers better outcome.

**Supplementary Information:**

The online version contains supplementary material available at 10.1186/s43163-023-00430-2.

## Background

India has been battling the COVID-19 crisis on multiple fronts and has recently witnessed a furious second wave causing a resurgence of a formerly lesser-known but highly fatal disease, rhino-orbital-cerebral mucormycosis (ROCM) [1]. India alone contributed to 81% of the cases of COVID-19-associated ROCM (CAM) during the second wave of COVID-19, although few case reports and retrospective studies of the same were published during the first wave of the pandemic as well [2–4]. CAM was reported from 18 different countries other than India; however, India is the largest contributor of the CAM cases reported within a duration of 3–4 months in 2021 [5].

Rhino-orbital-cerebral mucormycosis is an opportunistic, highly lethal invasive fungal infection of the nose and paranasal sinuses (PNS) that can invade the orbit and brain because of its propensity to invade blood vessels. The inhaled spores of rhizopus or rhizomucor, on reaching the nasal cavity and PNS, start to germinate if the environment is favorable and then invade the adjacent organs by causing tissue invasion, thrombosis, and necrosis [6]. Multiple risk factors have been incriminated for CAM, diabetes mellitus with or without uncontrolled sugars, steroid-induced hyperglycemia, and immunosuppression caused by the virus itself in COVID-19 infection [6, 7].

We intended to study the clinical profile of CAM patients and the COVID-19 patients who were suspected for mucor due to their clinical presentation, risk factors, management protocol, and their outcome. The information provided by this study may help us to recognize the early clinical features of CAM and to have a high index of clinical suspicion in the presence of typical symptoms and signs. This study may also help us to appropriately triage the patients, control the risk factors, confirm the diagnosis, establish staging, and initiate early protocol-based, multidisciplinary management.

## Methods

This is a single-center retrospective, descriptive observational study done in our COVID-19-designated hospital during the pandemic. Our hospital functioned as a full-time COVID designated hospital for over a period of 19 months, during which time we treated 17,000 COVID-19 in-patients, and we also diagnosed and treated 7 patients with COVID-associated mucormycosis.

### Sampling method

All the lab-confirmed cases of COVID-associated rhino orbital mucormycosis (CAM) and the clinical suspects of mucor managed in our hospital from april 2020 to July 2021**.**

We retrieved the medical records of all the lab-confirmed CAM cases and the clinical suspect cases of mucor that were managed in our hospital during the above-mentioned period. The medical records were retrieved after obtaining appropriate permissions from the concerned authorities.

The data regarding their demographics, clinical presentation, risk factors, signs and symptoms, COVID-19 treatment history, microbiological tests, histopathology reports, radiological investigations, endoscopic surgical findings, medical treatment, and follow-up endoscopy were recorded in a proforma. The follow-up of all CAM and the suspected cases of ROCM who were negative for mucor were contacted over the telephone to know their current status.

COVID-19 infection was diagnosed based on RT-PCR (real-time polymerase chain reaction) testing done in our institute and computed tomography scan of the thorax was done to know their COVID-19 pneumonia severity status. The COVID-19-positive patients were treated appropriately according to the guidelines issued by MOHFW, India (Ministry of Health and Family Welfare). The cases of cam were diagnosed as mucormycosis based on their clinical presentation and symptoms, such as (1) black necrotic turbinates, (2) blood-tinged nasal discharge and facial pain, (3) periorbital or perinasal swelling with discoloration, (4) ptosis, proptosis of an eyeball with ophthalmoplegia, and (5) multiple cranial nerve palsies.

Those who were suspected of CAM with known risk factors underwent baseline investigations, such as complete blood count, blood sugar levels, liver function tests, renal function tests, ENT clinical examination, diagnostic nasal endoscopy, microbiological investigations, and computed tomography scans of the nose and paranasal sinus. If the sample from the Nasal cavity showed broad aseptate fungal hyphae branching at wide angles on KOH mount, it was considered positive for mucormycosis, and further culture was done to know the species. Tissue from the middle turbinate and the debrided tissue specimen was sent for histopathological examination, and if that showed pauci septate fungi with tissue invasion and angio invasiveness that is pathognomonic of rhizopus species causing mucormycosis. Cases confirmed of CAM underwent the management protocol as given by MOHFW.

### Operational criteria

#### COVID-19-associated mucormycosis

The occurrence of mucormycosis in an individual within a median of 24 days (range 8–90) of COVID symptoms or 12.5 days (1–49) days of ICU admission and 16 days (1–49) after corticosteroid prescription [8].

#### Mucor suspects

The COVID-19 patients who were on treatment for their COVID symptoms developed signs and symptoms suggestive of mucormycosis.

### Statistical analysis

The data obtained from the patients were entered into Microsoft excel and analyzed using SPSS version 16. The variables were summarized as mean and frequency, and percentages for qualitative data.

## Results

### Clinical profile of COVID-associated mucormycosis patients: (Table 1)

**Table 1:** Clinical profile of COVID-associated mucormycosis patients

Sl. No	Age/sex	Diabetic status at the time of diagnosis of CAM	COVID severity status	Steroid therapy: IV Dexa 8 mg once daily	Other coexisting risk factors	Symptoms at presentation	Mucor clinical staging	Treatment given (mucor)	Outcome
1	44/F	Uncontrolled/DKA	Moderate	4 days	Sepsis/acute renal failure	Eyelid and facial swelling, nasal discharge, crusting	Stage 2 Eschar of the facial skin, visual disturbances with complete loss of vision	Nil	Expired cardiac arrest, respiratory failure and multiorgan failure
2	71/M	Uncontrolled/DKA	Moderate	14 days	Hypertension CKD CAD	Eyelid and facial swelling, facial pain, nasal crusting	Stage 1 Nasal discharge Facial pain	IV ampho + Posa) 40 days Endoscopic sinus debridement	Expired after 1 month of treatment cardiac failure
3	52/M	Uncontrolled	Moderate	10 days	NIL	Eyelid and facial swelling, facial pain, peri orbital pain, head ache, nasal crusting	Stage 2 Eschar of the facial skin, visual disturbances with complete loss of vision	IV ampho + Tab. Posa 51 days Endoscopic debridement, orbital exenteration with total maxillectomy	Alive
4	63/M	Controlled	Moderate	10 days	HT	Eyelid and facial swelling, facial pain, nasal crusting	Stage 1	IV ampho + Tab. Posa 2 weeks ampho and 2 months Posa Endoscopic sinus Debridement	Alive
5	53/M	Uncontrolled/DKA	Mild	10 days	Sepsis/acute renal failure	Eyelid and facial swelling, pain around the eye with head ache, nasal crusting	Stage 3 Eschar of the facial skin and necrosis of the hard palate, visual disturbances with complete loss of vision	IV amphotericin 1 day	Expired due to sepsis and multiorgan failure
6	55/M	Controlled	Mild	10 days	NIL	Facial pain, nasal discharge, crusting	Stage 1	IV ampho + Tab. Posa 28 days 4 doses of Inj Ampho B retro bulbar Endoscopic sinus debridement	Alive
7	35/M	Uncontrolled	Mild	10 days	NIL	Pain around the eye with headache, nasal crusting	Stage 1	IV ampho for 5 days Endoscopic debridement	Alive

*Ampho* Inj. liposomal amphotericin B, *Posa* Posconazole, *DKA* Diabetic keto acidosis, *HT* Hypertension, *CKD* Chronic kidney disease, *CAD* Coronary artery disease

We diagnosed and treated 7 patients with CAM of varying clinical severity in our institute, six patients presented during the second wave of COVID-19 between May 2021 to August 2021 and 1 patient presented during the first wave in August 2020, 6 male and 1 female patient with a mean age of 45 years, between 35 and 71 years.

#### Probable risk factors for CAM

1. Diabetes: all seven patients diagnosed with CAM were diabetic, and 5 out of 7(71%) presented with uncontrolled diabetes mellitus at the time of diagnosis of CAM.
2. COVID status: all 7 CAM patients were COVID-positive, and all 7 were not vaccinated against COVID at the time of diagnosis of mucormycosis. All 7 developed CAM within a median duration of 7 days of diagnosis of COVID-19.
3. Steroid therapy: all 7 patients had been treated with Intravenous Dexamethasone 8 mg; 4 patients had moderate COVID pneumonia and required both steroid and oxygen therapy for a mean duration of 10.25 days, while 3 patients with mild COVID pneumonia were treated with steroids for an average duration of 9 days.
4. Antibiotics: all 7 patients were treated with broad-spectrum IV antibiotics for a mean duration of 7 days

#### Co-morbidities

Two patients had systemic hypertension, and one had both coronary artery disease and chronic kidney disease.

#### Clinical symptoms of mucormycosis

**Fig. 1:**
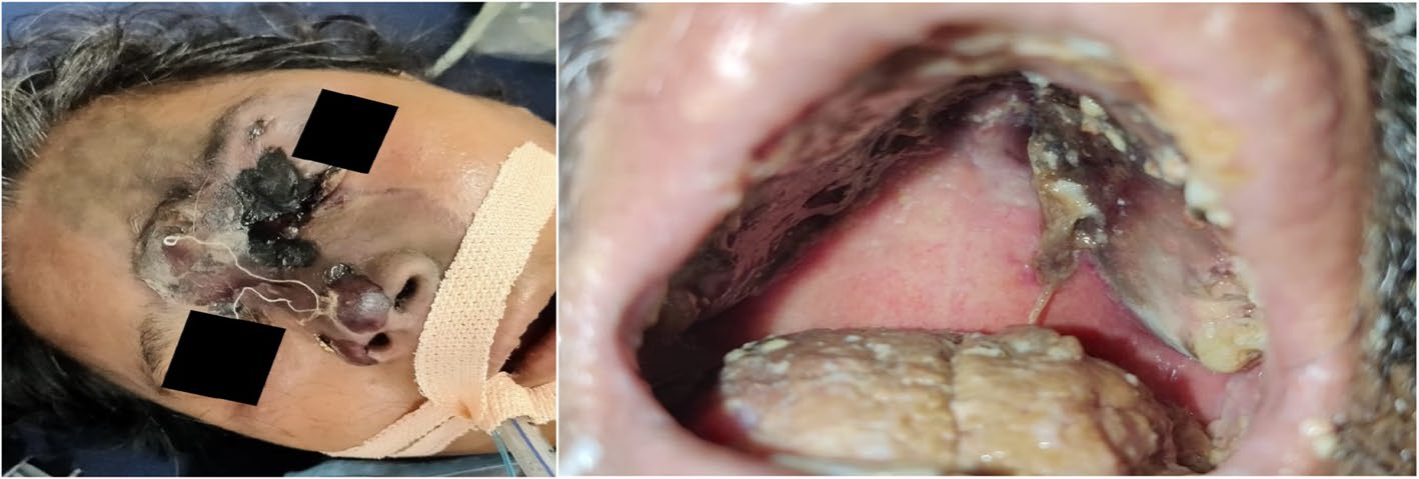
Showing A CAM patient with black eschar around the orbit, another patient with palatal necrosis with black crusts

During their course of treatment, 5 (71%)patients developed eyelid swelling, and facial swelling, 4(57%) patients developed facial pain, 3 (43%) had pain around the eye along with headache, all 7(100%) had nasal crusting, 2(28%) had paraesthesia of the face, 3(43%) developed eschar of the facial skin and necrosis of the hard palate (Fig. 1), and 4(57%) patients developed visual disturbances, and 3 (43%) patients had complete loss of vision.

#### On anterior rhinoscopy examination

**Fig. 2:**
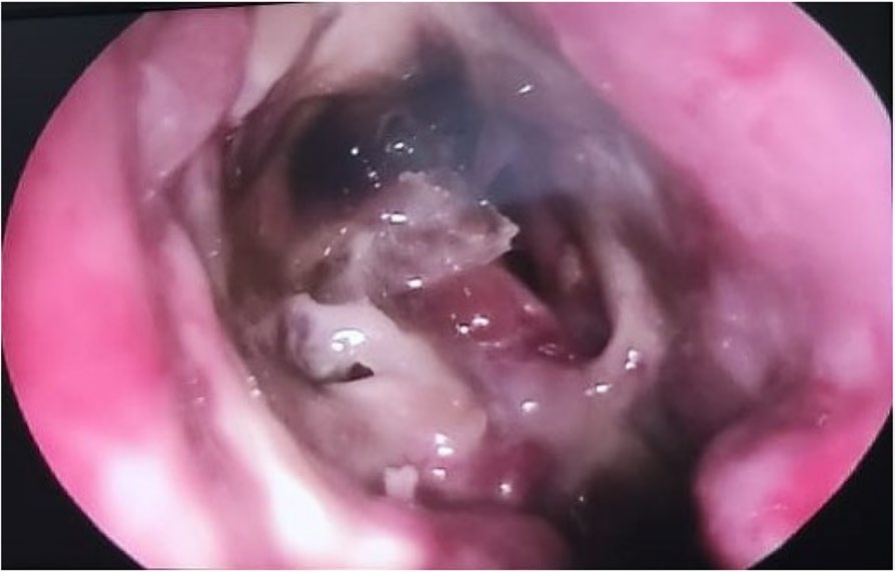
Nasal endoscopic picture showing black crusts around the necrosed middle turbinate in a CAM patient

All 7 CAM patients showed thick nasal crusting and mucopurulent discharge from bilateral middle meatus, and all 7 patients had black crusts around the necrosed middle turbinate (Fig. 2).

### Lab investigations

**Fig. 3:**
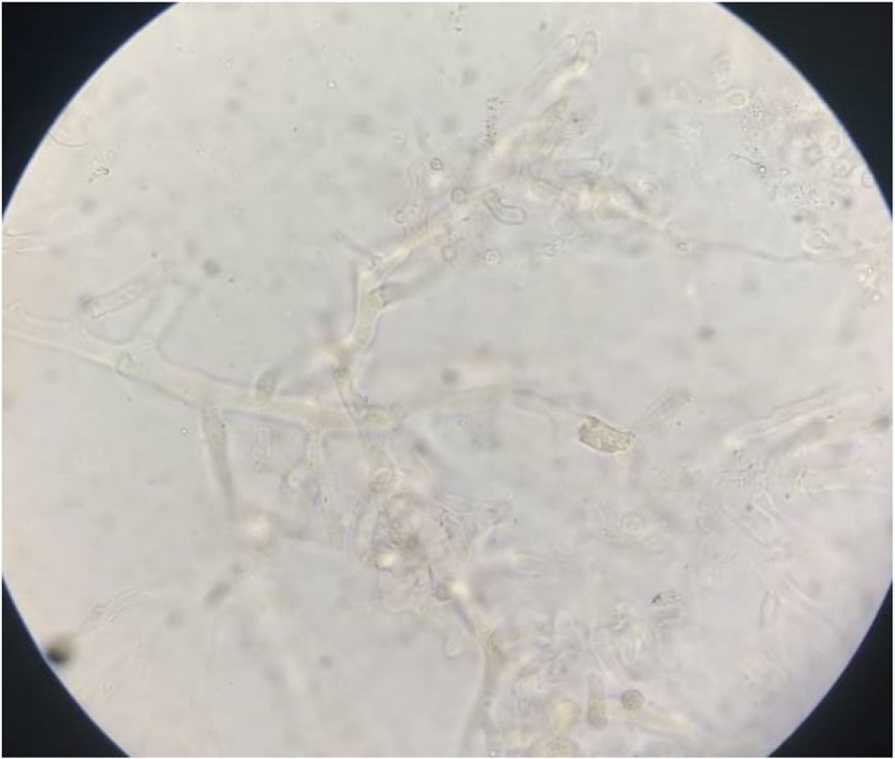
KOH mount showing broad aseptate hyphae branching at wide angles

All 7 CAM patients in our study underwent nasal swabs that were taken from the middle turbinate/middle meatus and sent for microbiological investigation. All 7 nasal swabs were KOH positive, showing broad aseptate fungal hyphae branching at wide angles suggestive of Mucor species (Fig. 3). The tissue from the middle meatus was sent for histopathological investigation that showed pauci septate, necrotizing fungal inflammation with angioinvasion suggestive of mucormycosis.

#### Radiological investigations

**Fig. 4:**
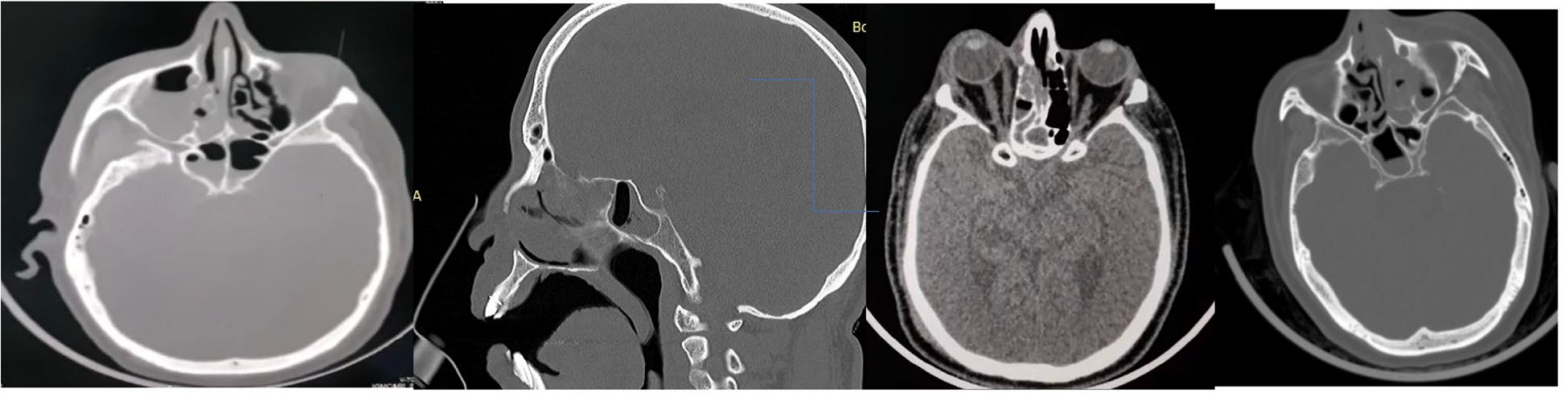
CT PNS of CAM patients showing involvement of maxillary, ethmoid and sphenoid sinuses, palatal necrosis, and skull base erosion

All 7 CAM patients underwent computed tomography (CT) scan of the nose and paranasal sinus that showed involvement of maxillary and ethmoids sinuses in all 7 (100%) patients (Fig. 4), 6 (86%) patients had sphenoid, and 4 (57%) showed frontal sinus involvement.

Maxillary sinus involvement varied from extensive mucosal involvement to hypodense fungal mass and bony erosions. The ethmoidal sinus also showed similar findings. Sphenoid and frontal sinuses showed hypodense fungal lesions in 2 patients, and frontal sinuses showed minimal mucosal involvement.

The orbit was involved in 4 (57%) patients showing preseptal cellulitis, fat plane thickening, and proptosis, and in 1 patient had superior ophthalmic vein thrombosis.

### Clinical staging of the patients (Table 1)

Three (42.8%) patients had stage 1 disease involving the nose and paranasal sinuses only. Three (42.8%) patients had stage 2 disease involving the nose, paranasal sinuses, and the orbit. One (14%) patient had stage 3 disease involving the sinuses, orbit, and brain.

#### Treatment

##### Surgical treatment

Four (57%) patients underwent endoscopic debridement of the paranasal sinuses, one (14%) underwent endoscopic debridement and orbital exenteration and two patients had extensive clinical disease involving the sinus, palate, and orbit, and their general condition was very poor for general anesthesia and surgical management.

#### Medical treatment

Four CAM patients were started on injection amphotericin B (liposomal) at a dose of 5 mg/kg for 2 weeks, as per protocol, in 5% dextrose over 3 h, followed by post-hydration. The anti-fungal treatment was then stepped down to oral Posaconazole 300 mg once daily for two months. One patient was treated with retrobulbar injection of amphotericin B in the dose of 3.5 mg/ml for 3 days in view of the loss of vision in addition to systemic Amphotericin B; however, this patient did not have an improvement in vision. None of our patients developed allergic/adverse reactions to Amphotericin B, nor did they develop acute kidney injury.

#### Morbidity and the outcome

The average duration of hospital stay was between 40 and 100 days. 2(28%) patients had extensive mucormycosis; their average duration of hospital stay was 4 days, and both expired. 1 patient expired after 30 days of discharge from the hospital post endoscopic debridement and medical therapy. Four (57%) out of 7 patients with CAM are alive till 16 months of follow-up.

#### Clinical profile of mucormycosis suspects

Fourteen patients were clinically suspected of having mucormycosis while being treated for their COVID-19 symptoms and were referred to the ENT department for management. This mucor suspect group had 9 female and 5 male patients with a mean age of 49.5 years; the range was 20 to 73 years.

### Probable risk factors for CAM

1. Uncontrolled diabetes: 9 were known diabetic patients, 6 had uncontrolled diabetes mellitus, and 3 had sugars under control at the time of clinical suspicion of CAM.
2. COVID-19 status: all 14 were not vaccinated for COVID-19 and were in hospital care at the time of suspicion for CAM. Ten patients required oxygen therapy for their COVID status for an average 18 days.
3. Steroid therapy: 12 patients were treated with steroids for their COVID-19 symptoms for an average duration of 10.3 days.
4. Antibiotics: all 14 patients were treated with IV antibiotics for an average duration of 15.3 days

### Comorbidities

Three patients had systemic hypertension and one with COPD and respiratory failure.

### Clinical symptoms suggestive of CAM

Six patients had developed mild eyelid swelling, and pain around the eye, five had facial pain and facial swelling, and four had nasal discharge.

### Radiological investigations

CT scan of PNS revealed normal CT PNS in 4, 7 had minimal mucosal thickening in the maxillary sinus, 3 had minimal mucosal thickening in the frontal sinus, 2 in ethmoidal sinus and 1 in the sphenoid sinus.

### Lab investigations

Seven suspect patients underwent nasal swabs for KOH mount and PAS stain, which were negative for mucor; the other 7 patients were under observation; their risk factors were brought under control, and they were discharged since they did not develop symptoms of mucormycosis.

### Outcome

Among the 14 suspects, 2 were COVID-negative and were discharged from the hospital, 3 patients expired due to COVID-19 complications, and 9 patients are alive till date.

## Discussion

The true incidence and prevalence of ROCM in India have not been studied so far; prior to COVID-19, a prevalence of 14 cases per 100,000 individuals was estimated by employing a computational-model-based method [8–11]. During the second wave of COVID-19 in India, a rough estimate of over 20,000 cases were reported within a short span of 3 to 4 months, and this has been linked to the COVID-19 variant B.1.617.2 (Delta variant) [12, 13]. Although CAM cases have been reported from 18 other countries, India has reported the highest number of cases till now [13]. In our hospital, we treated 7 CAM patients out of 17,000 COVID-19 inpatients, and the prevalence is 0.04%; however, this may not reflect the burden in the community as this is a hospital-based study. The concerned states and union territory governments have taken imminent measures to bring the situation under control by issuing guidelines and procuring the drugs required for treatment. The Indian Council of Medical Research (ICMR) released guidelines for the screening, diagnosis, and management of mucormycosis in patients with COVID-19, and the ministry of health and family welfare (MOHFW) India, has made this rare disease notifiable [14, 15].

### Demographic profile

In India, the Northern part of India reported more cases of CAM than the southern part of the country, simply reflecting the burden of diabetes mellitus being 67% in North India compared to 22% in the south [14, 15]. We treated 7 patients of CAM, 6 males and 1 female patient, age range between 35 and 71 years (mean 45 years), and this demographic pattern of men being more affected than women is reflected in other studies done in India as well [16].

### The probable risk factors for COVID 19-associated mucormycosis

#### Diabetes mellitus

The presence of diabetes mellitus is considered a major predisposing factor for mucormycosis even prior to the COVID-19 era [3, 4] with an odds ratio of (OR) 2.49; 95% CI 1.77–3.54; *p* < 0.001 [17]. Similarly, in a study done by Sen et al., multicentric collaborative study of 2826 patients, 78% were diabetic, and 44% were uncontrolled or had diabetic ketoacidosis (DKA) at the time of diagnosis CAM [7]. In another meta-analysis by author Jeong et al., 70% of patients with rhino-orbital–cerebral mucormycosis had diabetes as a risk factor, and this also reflects in other studies as well [15, 17]. The review article by Hoenigl et al. states that specifically in India, diabetes was a more predominant risk factor when compared to the rest of the world [95%] of 42 patients in India vs. 26 [68%] of 38 from other countries, making DM as a significant risk factor [5].

Uncontrolled diabetes mellitus/hyperglycemia seems to be causing immune dysregulation, which predisposes these patients to develop fungal co-infection, especially mucormycosis [17]. Uncontrolled sugars also help the organism to thrive in a conducive environment by increasing blood ferritin levels through various methods [18]. In our study, all 7 patients were known diabetics, and 5 patients (71%) presented with high sugars at the time of diagnosis with mucormycosis.

#### COVID status

All 7 CAM were positive for COVID-19 and were not vaccinated against COVID-19 at the time of their diagnosis. There seem to be few factors that favor the propagation of the mucorale spores in these patients, hypoxic environment, steroid-induced hyperglycemia, acidotic status [diabetic ketoacidosis (DKA) and sepsis-related metabolic acidosis], and immunosuppression, all interact and predispose these patients for a florid growth of ROCM [19–21].

#### Steroid therapy

Currently, clinical trials have conclusively shown that corticosteroids are the only medication to be effective in the treatment of COVID-19, but an indiscriminate and irrational usage of the same has been incriminated as a potential risk factor for developing CAM [19–21]. The updated national guidelines have the formulation and dose recommended for clinical usage for a duration of 5–10 days [22].

In our study, all 7 patients were treated with systemic corticosteroids for a duration of 9 to 10 days. Four CAM patients (57%) patients who are alive currently, the steroids were immediately discontinued once CAM was diagnosed in them and the sugars were brought under control. The usage of systemic steroids seems to exaggerate the underlying glycemic control as well as impede the body’s immune system [23, 24]. Optimizing steroids in the setting of CAM seems to offer the best possible outcome in these patients.

#### Antibiotics

It is felt by many experts, that the overuse of antibiotics during COVID-19 management suppresses the normal bacterial flora and facilitates the establishment and invasion of fungi or a fungal co-infection [25, 26]. Another systematic review by Langford et al. also supported the fact that overuse of broad-spectrum antibiotics was common (74.6%) in cases of COVID-19 with mucormycosis [25–28]. All our CAM patients were treated with broad-spectrum antibiotics for an average duration of 7 days and the mucor suspects for a duration of 15.3 days.

### Comorbidities

We observed in our study that the presence of other co-morbid conditions, like acute renal failure and sepsis in 2 CAM patients, influenced the initiation of inj amphotericin B treatment, their prognosis was very poor, and both patients expired.

### Clinical features of mucormycosis

In our study, 4 (57%) patients presented with nasal discharge and eye swelling, which were the commonest clinical features as in other studies [12, 16, 18]. On anterior rhinoscopy examination, thick, blackish nasal crusts and necrosed middle turbinate were seen in all 7 CAM patients, as seen in other studies as well [29]. The tissue biopsy of the mucosa from the middle turbinate was positive for Mucormycosis as in other studies [12].

All the mucor suspects lacked the typical clinical features and signs of mucormycosis, and the nasal examination also did not show features suggestive of mucor as also the radiological investigations.

### The clinical staging of CAM

There exists no sound staging system to categorize the disease severity of ROCM. The most commonly followed clinical staging is.

- Stage I: infection of the nasal mucosa and sinuses.
- Stage II: orbital involvement (orbital apex syndrome, superior orbital fissure syndrome).
- Stage III: cerebral involvement [30].

In our case series of CAM, 3 (42.8%) patients had stage 1 disease, 3 (42.8%) patients had stage 2 disease involving the nose, para nasal sinuses, and the orbit, and 1 (17%) patient had stage 3 disease.

Six of our CAM patients belonged to the rhino orbital mucormycosis (ROM), which was the commonest form of CAM reported from India, and 1 patient had rhino orbital cerebral mucormycosis that was seen in other studies as well [5, 6, 12, 16].

### Lab investigations

All the 7 CAM patients in our series were KOH positive for mucormycosis and were confirmed by histopathological studies as in other studies [4–6, 12].

### Radiological investigations

In our study, the maxillary and ethmoid sinuses were the most commonly involved sinuses (7/7), as seen in other studies, although the radiological findings varied according to the clinical staging [16, 20].

### Treatment

Four (57%) patients in our study had the early clinical diagnosis and therefore underwent surgical and medical treatment. Injection Amphotericin B as per protocol was initiated, and Tab. Posaconazole as a step-down treatment was followed as recommended by the MOHFW guidelines that were formulated based on the global guidelines given by the European confederation of medical mycology and mycoses study group and research consortium [31]. These 4 patients are alive after more than 1 year of follow-up.

ROCM has a high mortality rate of 49% in diabetic patients even before the COVID era [9, 10, 12, 13, 28]. The new additional factor that seems to be influencing the prognosis is COVID itself. Few studies from India have reported high mortality in CAM patients who had severe COVID status [16, 32], whereas other studies like Sharma et al., had no mortality in their case series of 23 CAM patients [33]. The mortality rate in our study is 42.8% which was similar to that seen in other studies as well [3, 5]. But in an actual sense, the mortality rate cannot be comparable from one center to another because it largely depends upon a case-to-case basis. Multiple factors can influence the outcome in CAM like early clinical diagnosis, COVID status, rapid control of uncontrolled sugars, optimization of steroids, and early surgical debridement and anti-fungal therapy.

## Limitations of our study

1. Our study comprised only 7 CAM patients, and we cannot arrive at a causal-to-effect relationship.
2. This descriptive study has less number of cases, and therefore our results cannot be extrapolated to the results of studies in other geographical areas.

## Conclusion

In the current setting, our descriptive study can say that the triad of uncontrolled sugars, irrational corticosteroid use, and the presence of COVID-19 infection have a role in causing rhino orbital mucormycosis. We suggest that the clinical suspicion of ROM should be very high in patients with these risk factors; however, optimization of the same, early identification, and early surgical debridement followed by medical treatment would be the key to a better prognosis.

## Supplementary Information

**Additional file 1.**

## Data Availability

The dataset supporting the conclusions of this article is included within the article and its Additional file.
